# Molecular basis for the sensitivity of TRP channels to polyunsaturated fatty acids

**DOI:** 10.1007/s00210-018-1507-3

**Published:** 2018-05-08

**Authors:** Marc Riehle, Dmitry Tsvetkov, Björn-Oliver Gohlke, Robert Preissner, Christian Harteneck, Maik Gollasch, Bernd Nürnberg

**Affiliations:** 1 Department of Pharmacology and Experimental Therapy, Institute of Experimental and Clinical Pharmacology and Toxicology, Eberhard Karls University Hospitals and Clinics and Interfaculty Center of Pharmacogenomics and Drug Research (ICePhA), Wilhelmstrasse 56, 72074 Tübingen, Germany; 2 Experimental and Clinical Research Center (ECRC), a joint cooperation of the Charité University Medicine and Max Delbruck Center for Molecular Medicine in the Helmholtz Association, Lindenberger Weg 80, 13125 Berlin, Germany; 3 Medical Clinic for Nephrology and Internal Intensive Care, Charité Campus Virchow Klinikum, Berlin, Germany; 4 Structural Bioinformatics Group, Institute for Physiology, Charité - University Medicine Berlin, Berlin, Germany

**Keywords:** *Drosophila*, TRPC channels, Polyunsaturated fatty acids, TRP channels, Ca^2+^ influx

## Abstract

Transient receptor potential (TRP) channels represent a superfamily of unselective cation channels that are subdivided into seven subfamilies based on their sequence homology and differences in gating and functional properties. Little is known about the molecular mechanisms of TRP channel regulation, particularly of the “canonical” TRP (TRPC) subfamily and their activation by polyunsaturated fatty acids (PUFAs). Here, we analyzed the structure-function relationship of *Drosophila* fruit fly TRPC channels. The primary aim was to uncover the molecular basis of PUFA sensitivity of *Drosophila* TRP-like (TRPL) and TRPgamma channels. Amino acid (aa) sequence alignment of the three *Drosophila* TRPC channels revealed 50 aa residues highly conserved in PUFA-sensitive TRPL and TRPgamma channels but not in the PUFA-insensitive TRP channel. Substitution of respective aa in TRPL by corresponding aa of TRP identified 18 residues that are necessary for PUFA-mediated activation of TRPL. Most aa positions are located within a stretch comprising transmembrane domains S2–S4, whereas six aa positions have been assigned to the proximal cytosolic C-terminus. Interestingly, residues I465 and S471 are required for activation by 5,8,11,14-eicosatetraynoic acid (ETYA) but not 5,8,11-eicosatriynoic acid (ETI). As proof of concept, we generated a PUFA-sensitive TRP channel by exchanging the corresponding aa from TRPL to TRP. Our study demonstrates a specific aa pattern in the transmembrane domains S2–S4 and the proximal C-terminus essential for TRP channel activation by PUFAs.

**Electronic supplementary material:**

The online version of this article (10.1007/s00210-018-1507-3) contains supplementary material, which is available to authorized users.

## Introduction

Transient receptor potential (TRP) channels are forming a superfamily of unselective cation channels present throughout the animal kingdom and humans. Based on sequence homology and differences in gating and regulation, TRP channels are grouped into several subfamilies (transient receptor potential canonic (TRPC), TRPV, TRPM, TRPML, TRPA, TRPP, and TRPN) (Harteneck et al. 2000; Montell et al. 2002; Minke 2010). Biological functions of TRP channels in mammals are exceptionally diverse and include the regulation of the ionic homeostasis, vascular tone, nociception, or cell death (Hardie 2011). In the *Drosophila* fruit fly, the TRPC subfamily comprises three members, i.e., TRP, TRP-like (TRPL), and TRPgamma (Hardie 2011). TRP and TRPL are expressed at high levels in photoreceptor cells functioning as main light-sensitive Ca^2+^ channels participating in phototransduction (Hardie and Minke 1992; Hardie 2014; Reuss et al. 1997). The third member of the subfamily, i.e., TRPgamma, shows a much broader expression spectrum and plays a role in neurosecretion and motility fine tuning (Xu et al. 2000; Jors et al. 2006; Wicher et al. 2006; Akitake et al. 2015). In humans, two members of TRPC channels have been implicated in kidney disease. Mutated TRPC6 channels cause focal segmental glomerular sclerosis (FSGS), with most mutations resulting in a gain-of-function (GOF) phenotype (Winn et al. 2005; Reiser et al. 2005; Riehle et al. 2016). GOF mutations of TRPC6 lead to increased calcium entry and podocyte cell death (Winn et al. 2005). Recently, GOF mutations producing enhanced TRPC5 activity have also been found to drive progressive chronic kidney diseases (Zhou et al. 2017). Thus, insights into the activation mechanisms of TPRC channels can foster our molecular understanding of the pathogenesis of genetic and acquired forms of FSGS and other chronic kidney diseases allowing the development of novel concepts for pharmacological intervention.

All TRP channels contain six transmembrane domains flanked by cytosolic N- and C-termini. Within the TRP family in *Drosophila*, TRP shows the highest selectivity for Ca^2+^ which is determined by aspartate 621 in the pore region (Liu et al. 2007), whereas TRPL display only a low ion selectivity (Reuss et al. 1997). TRPL and TRPgamma are activated in cells following a receptor-induced PI-response, i.e., phospholipase Cbeta (PLCbeta)-dependent hydrolysis of phosphatidylinositol 4,5-bisphosphate (PIP_2_) to inositol 1,4,5-trisphosphate (IP_3_), H^+^, and diacylglycerol (DAG). However, the exact mechanism of TRP channel activation by this pathway remains an unresolved mystery (Hardie 2014). Although the involvement of storage-dependent processes via IP_3_-receptors has been excluded (Hardie and Raghu 1998; Raghu et al. 2000a), DAG appears to play a major role as it triggers gating of these *Drosophila* TRP channels (Raghu et al. 2000b). Interestingly, additional endogenous lipids such as polyunsaturated fatty acids (PUFAs) also activate TPRL and TRPgamma (Chyb et al. 1999; Estacion et al. 2001; Parnas et al. 2009; Jors et al. 2006). Experimental data demonstrating structural elements responsible for transmitting lipid-induced stimulation are still missing for almost all TRP channels, despite specific lipid binding sites have been hypothesized enabling TRPV1 channel activation (Taberner et al. 2015). The purpose of the present study was to identify amino acid (aa) residues mediating PUFA-induced activation of *Drosophila* TRP channels. Our analyses identify 18 aa residues that are crucial for PUFA-mediated activation of TRPL. Twelve out of these 18 aa residues are localized in a stretch comprising the transmembrane domains S2–S4, whereas six aa were found in the proximal cytosolic C-terminus. We also identified two residues that allow discrimination of the channel sensitivity domains S2–S4 region together with the proximal C-terminus(ETYA) and 5,8,11-eicosatriynoic acid (ETI). We propose that aa residues within the transmembrane hyc 5?> of TRPL protein, required for PUFA-induced activation. This represents a unique feature within the TRPC channel subfamily.

## Materials and methods

### Materials

ETYA and ETI were purchased from Sigma-Aldrich (Germany) and dissolved in dimethyl sulfoxide (DMSO; Sigma-Aldrich, Germany). ETYA and ETI solutions were prepared as 20 mM stock solutions and frozen as small aliquots. To prevent oxidation of ETYA, the solvent was purged with an inert gas before use. Chemicals obtained from Sigma-Aldrich (Germany) were of the highest analytical grade. Both ETYA and ETI were applied at supramaximal effective concentrations (40 μM) (Jors et al. 2006; Chyb et al. 1999). DMSO concentration in the assays did not exceed 0.2%.

### Cell culture and transfection

HEK293 cells were cultured in a medium supplemented with Earl salts (PAA, Pasching, Austria), 10% FCS (PAA), 4 mM l-glutamine (PAA), 100 U/ml penicillin (PAA), and 100 μg/ml streptomycin (PAA) in humidified atmosphere at 37 °C and 5% CO_2_. After seeding, cells were transfected with 2 μg plasmid DNA coding for wild type (WT) or mutant TRPC isoforms using calcium phosphate, as previously described (Riehle et al. 2016). In brief, the transfection mixture contained 2 μg DNA; 112 μl H_2_O; 125 μl 2× BBS buffer containing 50 mM BES, 280 mM NaCl, and 1.5 mM Na_2_HPO_4_; and 12.5 μl CaCl_2_ (2.5 M). The medium was exchanged after 5–6 h and the cells were used for experiments 20–48 h after the transfection.

### Site-directed mutagenesis

Plasmid DNA coding for TRP, TRPL, or TRPgamma channels C-terminally fused to YFPs was used as the temf to generate the TRPL and TRP mutants, as previously reported (Riehle et al. 2016). We used the cDNA coding for TRP, TRPL, and TRPgamma as previously described (Jors et al. 2006). For site-directed mutagenesis, plasmids were amplified by oligonucleotides (custom made by Biomers, Ulm, Germany) harboring the sequences coding for the planned mutation (Supplementary Tables 1, 2, and 3). Amplification was performed using Phusion DNA Polymerase (Biozym, Hess. Oldendorf, Germany) using the temperature time protocol 98 °C for 30 s, 98 °C for 10 s and 71 °C for 420 s 18 times, and 72 °C for 600 s. Template DNA was removed by digestion using DpnI. The amplified DNA was used for the transformation by high efficiency-competent *Escherichia coli* NEB 5α (New England Biolabs, Ipswich, MA, USA). Plasmid DNAs from clones were isolated and sequenced. For the transfection, large-scale DNA preparations were performed using NucleoBond Xtra Maxi Kits (Macherey-Nagel, Dueren, Germany).

### Cell surface biotinylation

HEK293 cells were seeded in 94-mm dishes containing 1.4–2.9 million cells/dish and washed with the ice-cold PBS (pH = 8) 40–48 h after a transfection at 95% confluence. The composition of PBS (in mM) was 137 NaCl, 2.7 KCl, 8.1 Na_2_HPO_4_, and 1.47 KH_2_PO_4_. Cell surface biotinylation was carried out for 30 min at 4 °C by the addition of 0.5 mg/ml EZ-Link-Sulfo-NHS-LC-Biotin in PBS (pH 8) with regular panning. The deactivation of excess biotin was carried out by incubation with 100 mM glycine in PBS (pH = 7.4) (Riehle et al. 2016). Subsequently, the cells were resuspended with PBS (pH = 7.4), combined, and pelleted at 800×*g* for 5 min and RT (Table Centrifuge 5430, Eppendorf AG, Hamburg, Germany). The supernatant was discarded and the cells were resuspended in ice-cold lysis buffer. The lysis was continued by 2 × 4 ultrasound bursts and incubation on ice for 15 min. The removal of cell debris was carried out by centrifugation at 1000×*g* for 10 min at 4°. The documentation was carried out on the Luminescent Image Analyzer Versa-Doc-MP-4000-System®. For the quantification, the Image-Lab software was used (BioRad, Version 3.0). The detection of the channel proteins was performed via the YFP fusion portion by means of a GFP antibody. The isolation of the biotinylated samples was carried out via avidin-agarose beads using a spin-column system (Thermo Fischer Scientific GmbH, Schwerte, Germany).

### Intracellular Ca^2+^ measurements

For measurements of intracellular Ca^2+^ concentrations, HEK293 cells were exposed to sterile and uncoated coverslips (Riehle et al. 2016). After transfection and further incubation at 37 °C for 36–72 h, the cells were loaded for 30 min at 37 °C in standard HEPES buffer with 2 μM Fura-2-AM. Excess unrecorded Fura-2-AM and Fura-2 were removed by re-incubation in fresh HEPES buffer. The buffer was composed (in mM) of 138 NaCl, 6 KCl, 1 MgCl_2_, 2.5 CaCl_2_, 5.5 glucose, and 10 HEPES (adjusted to pH 7.4 with NaOH). The cells were placed into the measuring chamber, covered with 900 μl of new HEPES buffer, and prevented from drying out. Subsequently, the alternating measurement was started at 340/380 nm at room temperature. For the evaluation, at least 60 cells were selected from the field of view of the stably expressing cell lines. The identification of transfected cells was carried out via YFP fusion portion of the channel proteins at 474 nm. For normalization of expression levels, only cells from a defined range of relative fluorescence intensities have been selected. The background correction was performed by measuring a region containing no cells. The measurements were carried out on an inverted microscope AxioVert 100 with an objective FLUAR 10×/0.5 (Carl-Zeiss AG, Oberkochen, Germany). As the light source, we used a xenon vapor lamp with a monochromator to select the required excitation wavelength (Polychrom V, Imaging Control Unit ICU, FEI GmbH, Munich). The signal was detected using a charged coupled device camera (CCD, Sensicam, FEI GmbH, Munich, Germany).

### Generation of a tetrameric TRPL model

On the basis of solved tridimensional (3D) structures of TRPV1 and TRPM4, 3D model of TRPL was generated as previously reported in detail (Riehle et al. 2016). Briefly, transmembrane regions were aligned by the annotated helices. Additionally, the flexible regions (loops) as well as C- and N-terminus were separately aligned with Clustal Omega (Zimmermann et al. 2017). All parts were assembled to an overall alignment. Modeller version 9.14 was used to construct the 3D structure of TRPL (Webb and Sali 2016). The pipeline was built up by self-defined python scripts, for which all atom types, except of water molecules, were considered. To create the homo-tetrameric structure, the model was defined by special restrains to the c-alpha atoms to ensure the identity of subchains. The model was cleaned with respect to structural disorders, residue connectivity, and missing atoms in side chains and the backbone. For this purpose, Discovery Studio 4.1 was used (Discovery Studio; Accelrys Inc.; https://www.accelrys.com/dstudio). The final step comprises energy minimization to clean up psi/phy-angles inside the model.

### Statistical analysis

All data are expressed as the mean ± standard error of the mean (SEM). Statistical significance was determined by unpaired *t* test using Origin software (OriginLab, Northampton, MA, USA) (Riehle et al. 2016).

## Results

**Fig. 1:**
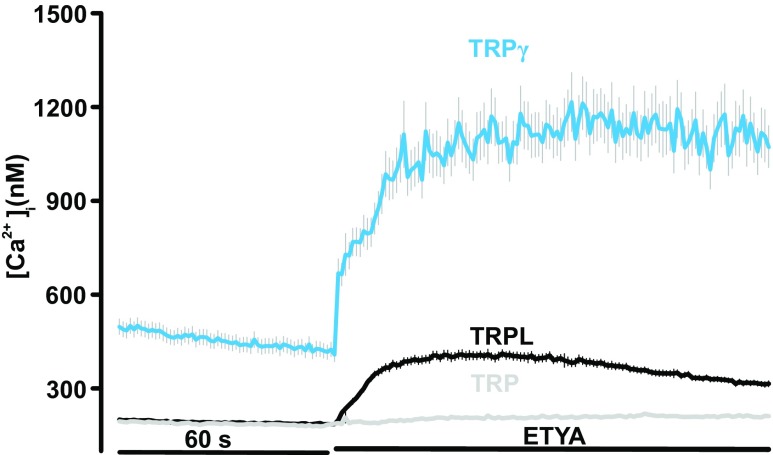
Functional characterization of the PUFA-induced activation of TRPgamma, TRP, and TRPL channels. Changes in intracellular calcium concentration were measured in Fura-2-loaded HEK293 expressing TRP (*n* = 1292), TRPL (*n* = 1918), and TRPgamma (*n* = 204) in response to application of 40 μM ETYA. Mean traces ± SEM are shown

We determined the effects of the prototypical PUFA ETYA (40 μM) on cytosolic calcium concentrations in HEK293 cells transfected with the three different members of the *Drosophila* TRPC channel subfamily. The expression of the TRPC protein in transfected HEK293 cells was confirmed by immunoblot analysis (Supplementary Fig. 1). Figure 1 shows that ETYA application increased free intracellular calcium concentration in HEK293 cells expressing TRPL and TRPgamma but not TRP. The difference in the response led us to propose that in contrast to TRP channels, TRPL and TRPgamma harbor specific aa residues allowing PUFA-induced activation. To address this question further, we analyzed the aa sequence identity of the three ion channels TRPL, TRPgamma, and TRP.

### Amino acid sequence comparison of the three *Drosophila* TRPC channels

**Fig. 2:**
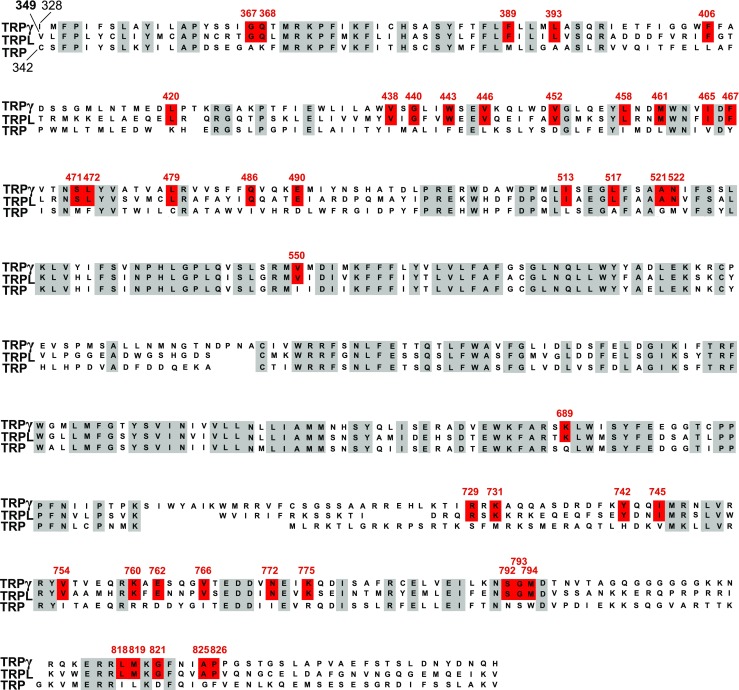
Sequence alignment of the *Drosophila* TRPgamma, TRPL, and TRP channels. Amino acid (aa) residues over all three TRPC channels have a gray background. The aa residues preserved in TRPL and TRPgamma but not in TRPL are highlighted in red. Numbered amino acid residues correspond to the sequence of TRPL channel from *Drosophila*

Figure 2 shows an aa sequence alignment of TRPgamma, TRPL, and TRP covering a stretch from the transition of the cytosolic N-terminus to the first transmembrane segment (S1) up to the proximal cytosolic C-terminus. Due to the importance of multimerization for channel function, more distal cytosolic regions of TRP channels were not examined. Comparison of the sequences revealed 50 aa residues that are preserved in TRPL and TRPgamma but not TRP suggesting that they are involved in PUFA-sensitivity. They are mainly localized within transmembrane domains S2–4 and the proximal portion of the cytosolic C-terminus (Fig. 2). Five of them, i.e., G599, D630, E633, G645, and L663, were either close to or within the pore region, while a sixth residue, L833, was located in a more distant part of the C-terminus and therefore omitted from further consideration. Due to the large number of candidate aa residues, we initially narrowed down the search for the PUFAs activation site(s) via the functional characterization of multiple mutants. For this purpose, we substituted stretches of up to four identified aa positions in the TRPL-WT channel by the corresponding aa of the ETYA-insensitive TRP channel. Then, we measured changes of free intracellular Ca^2+^ concentrations of the TRP mutants in response to EYTA stimulation.

### Functional characterization of multiple mutated TRPL channels

**Fig. 3:**
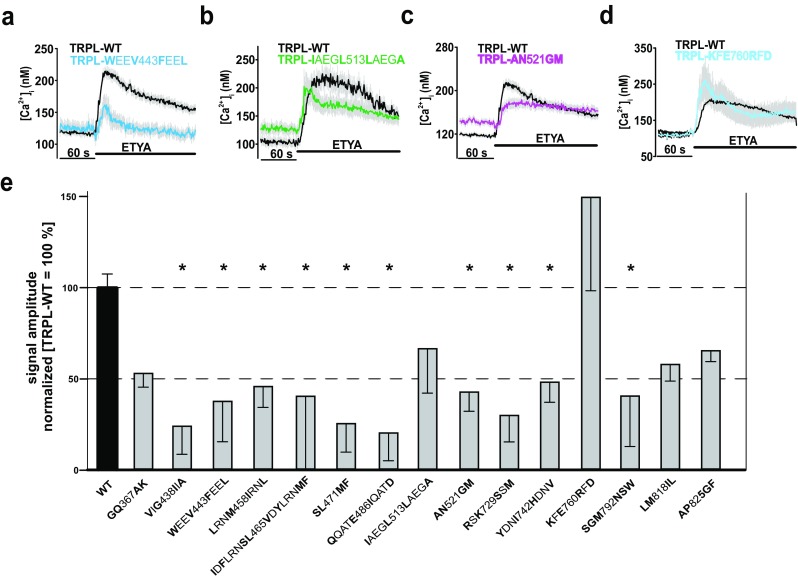
Functional characterization of TRPL multiple mutants. Changes in intracellular calcium concentration over time in response to application of 40 μM ETYA in Fura-2-loaded HEK293 expressing TRPL-WT compared to TRPL multiple mutants: TRPL-**W**EE**V**443**F**EE**L** (**a**), TRPL-**I**AEG**L**513**L**AEG**A** (**b**), TRPL-**AN**521**GM** (**c**), and TRPL-**K**F**E**760**R**F**D** (**d**). Mean traces ± SEM are shown. Statistical analyses of signal amplitudes obtained during at least three independent experiments are presented as bar graphs (**e**). Data are means of the normalized (WT = 100%) signal amplitudes ± SEM representing the tested multiple mutants. The analysis is based on the following cell numbers: **GQ**367**AK**, *n* = 207; **V**I**G**438**I**I**A**, *n* = 35; **W**EE**V**443**F**EE**L**, *n* = 60; **L**RN**M**458**I**RN**L**, *n* = 61; **I**D**F**LRN**SL**465**V**D**Y**LRN**MF**, *n* = 38; **SL**471**MF**, *n* = 148; **Q**QAT**E**486**I**QAT**D**, *n* = 99; **I**AEG**L**513**L**AEG**A**, *n* = 66; **AN**521**GM**, *n* = 109; **R**S**K**729**S**S**M**, *n* = 103; **Y**DN**I**742**H**DN**V**, *n* = 50; **K**F**E**760**R**F**D**, *n* = 48; **SGM**792**NSW**, *n* = 35; **LM**818**IL**, *n* = 144; and **AP**825**GF**, *n* = 203. *p ≤ 0.05. Substituted amino acid residues are marked in bold

For the functional characterization of the above-described multiple TRPL mutants, transfected HEK293 cells were stimulated with 40 μM ETYA. TRPL-WT cells served as controls. In this way, we examined a total of 15 different multiple mutants (Fig. 3e). This approach allowed us to identify regions important for ETYA action. For instance, in comparison to the response of TRPL-WT expressing cells, the calcium responses of cells expressing TRPL-**W**EE**V**443**F**EE**L** or TRPL-**AN**521**GM** displayed more than 50% reduction as determined by measuring signal amplitudes (Fig. 3a, c), whereas others like TRPL-**K**F**E**760**R**F**D**-expressing cells rather showed an increase in ETYA-dependent calcium responses (Fig. 3d). Mutants demonstrating only a small decrease in PUFA-induced stimulation of free cytosolic Ca^2+^ concentrations like TRPL-**I**AEG**L**513**L**AEG**A** (Fig. 3b) were excluded from further analysis (i.e., TRPL-**I**AEG**L**513**L**AEG**A**, TRPL-**K**F**E**760**R**F**D**, TRPL-**LM**818**IL**, TRPL-**AP**825**GF)**. To identify specific aa residues required for PUFA-induced activation, we generated TRPL single mutants and measured intracellular Ca^2+^ responses to ETYA application next (Supplementary Table 5).

### Functional characterization of TRPL single mutants

**Fig. 4:**
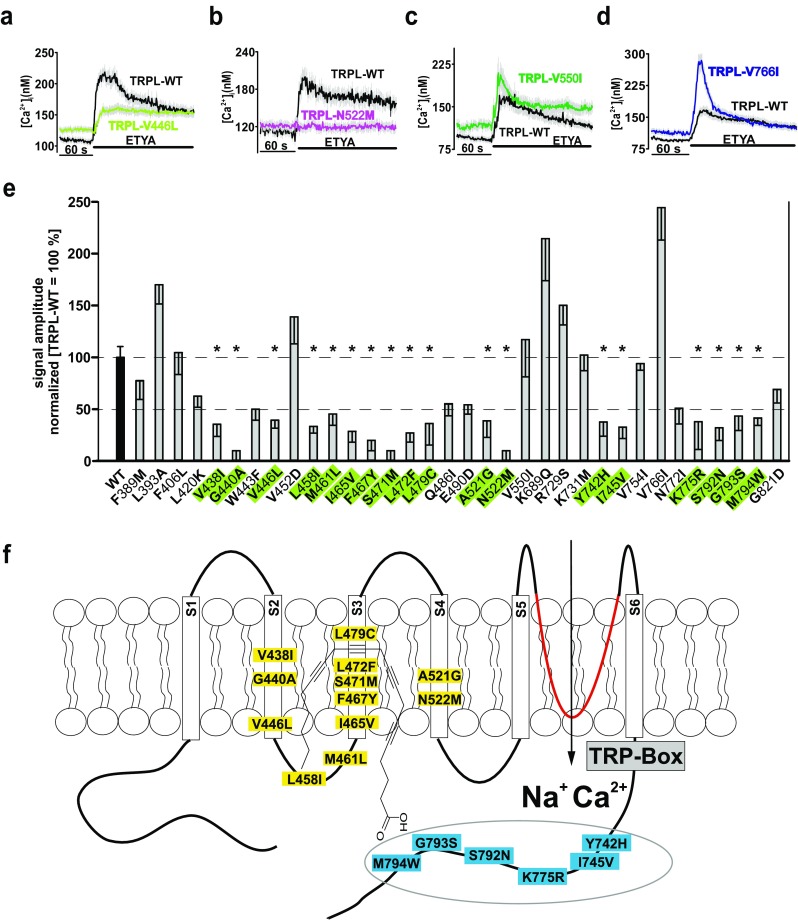
Functional characterization of TRPL single mutants. Changes in intracellular calcium concentration over time in response to application of 40 μM ETYA in Fura-2-loaded HEK293 expressing TRPL-WT compared to TRPL-**V**446**L** (**a**), TRPL-**N**522**M** (**b**), to TRPL-**V**550**I** (**c**), and to TRPL-**V**766**I** (**d**). Mean traces ± SEM are shown. Statistical analyses of data obtained during at least three independent experiments are presented as bar graphs (**e**). Data are means of the normalized (WT = 100%) signal amplitudes ± SEM representing the characterized 34 single mutants, in total, **F**389**M**, *n* = 249; **L**393**A**, *n* = 104; **F**406**L**, *n* = 214; **L**420**K**, *n* = 116; **V**438**I**, *n* = 135; **G**440**A**, *n* = 135; **W**443**F**, *n* = 103; **V**446**L**, *n* = 212; **V**452**D**, *n* = 50; **L**458**I**, *n* = 109; **M**461 **L**, *n* = 129; **I**465**V**, *n* = 96; **F**467**Y**, *n* = 171; **S**471**M**, *n* = 93; **L**472**F**, *n* = 152; **L**479**C**, *n* = 100; **Q**486**I**, *n* = 207; **E**490**D**, *n* = 204; **A**521**G**, *n* = 147; **N**522**M**, *n* = 136; **V**551**I**, *n* = 46; **G**645**A**, *n* = 189; **K**689**Q**, *n* = 54; **R**729**S**, *n* = 81; **K**731**M**, *n* = 128; **Y**742**H**, *n* = 62; **I**745**V**, *n* = 151; **V**754**I**, *n* = 113; **V**766**I**, *n* = 71; **N**772**I**, *n* = 49; **K**775**R**, *n* = 92; **S**792**N**, *n* = 105; **G**793**S**, *n* = 141; **M**794**W**, *n* = 207; **G**821**D**, *n* = 186. *p ≤ 0.05. Substituted amino acid residues are marked in bold. Two-dimensional schematic representation of the TRPL channel in the membrane with the amino acid residues that are necessary for PUFA-mediated activation of TRPL is shown (**f**)

Calcium responses of the corresponding single mutants gave a much more differentiated picture of the function of individual aa in the channel activation by ETYA. Our analysis allowed stratification of the 34 mutants into two different groups. In the first group, mutants such as TRPL-V446L showed a reduced Ca^2+^ influx of more than 50% (Fig. 4a, e) or even lacked any Ca^2+^ response to ETYA (e.g., TRPL-N522M) compared to TRPL-WT (Fig. 4b, e). The second group of mutants, including TRPL-V550I (Fig. 4c, e), displayed either a similar response or a larger signal amplitude (TRPL-V766I mutant) (Fig. 4d, e) compared to TRPL-WT. In all the experiments, overexpressed TRPL-WT-channels served as positive controls and mutants located within the ion pore region (i.e., **D**DF**E**631**V**DF**D** and D631V) served as negative controls (Supplementary Fig. 2). Therefore, 18 aa residues listed in the first group are identified to be involved in PUFA activation and assigned to regions lying between transmembrane domains S2–S4 (12 aa) and the proximal C-terminus (six aa) (Fig. 4e, f). To strengthen our findings, a phylogenetic sequence identity analysis of TRP, TRPL, and TRPgamma channels was carried out in ten insect species.

### Phylogenetic analysis of different insect species

**Fig. 5:**
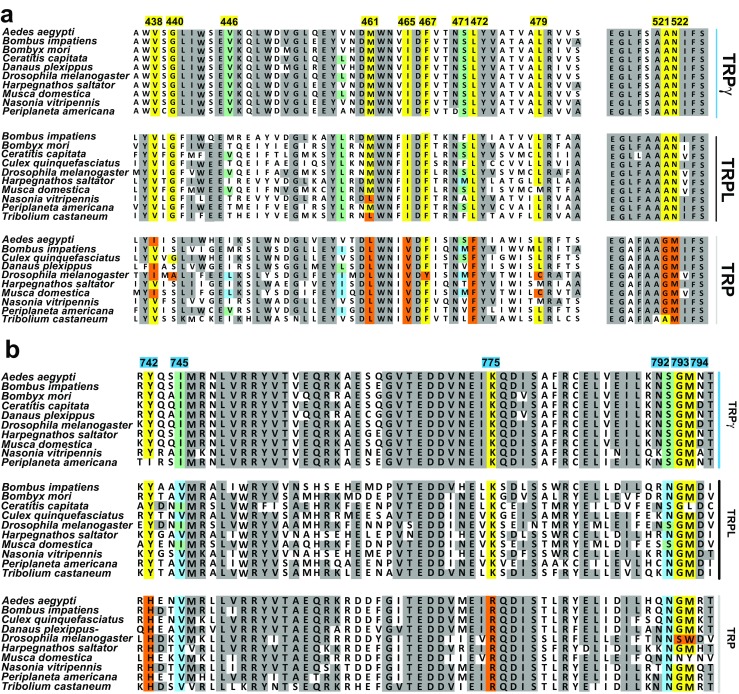
Phylogenetic analysis of TRPgamma, TRPL, and TRP channels in various insect species. A transmembrane segment (**a**) and cytosolic C-terminus (**b**) of TRPgamma/TRPL/TRP homologous sequence are shown. Numbered amino acid residues correspond to the sequence of TRPL channel from *Drosophila*. In yellow (TRPgamma/TRPL conserved) and orange (TRP conserved) are highly conserved positions, within the identified aa positions. In light blue (conserved to TRP) and light green (conserved TRPgamma/TRPL), aa positions that are not completely preserved throughout the channels

Figure 5 illustrates sequence alignments of stretches covering transmembrane regions S2–S4 (Fig. 5a) and the proximal cytosolic C-terminus (Fig. 5b) of TRPgamma/TRPL/TRP homologous sequences from ten different insect species (Supplementary Table 4). aa positions 465, 472, 522 (see Fig. 5a), and 775 (see Fig. 5b) are identical either in the PUFA-sensitive TRPgamma and TRPL channels on the one hand or within PUFA-insensitive TRP channel on the other hand across different species. This obvious identity of particular aa across species supports the idea of an essential role of these aa residuals for determining PUFA sensitivity.

### Influence of ETI on TRPL mutants

**Fig. 6:**
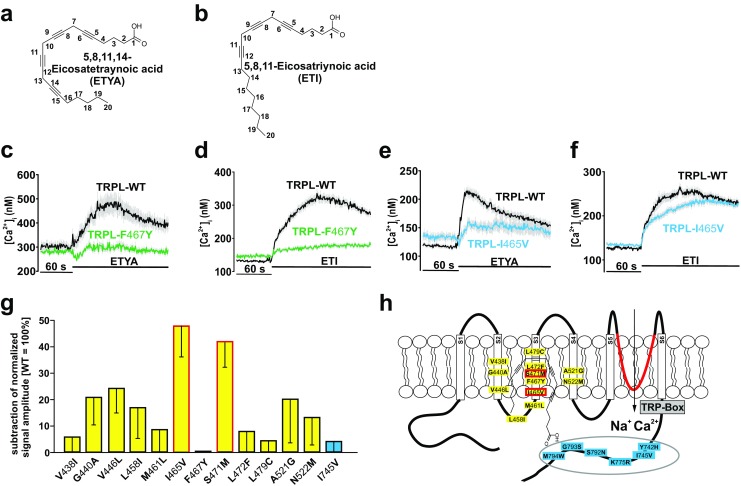
Functional characterization of different aa positions on ligand-specific effects of ETYA and ETI. Two-dimensional structures of ETYA (**a**) and ETI (**b**) are depicted. Changes in intracellular calcium concentration over time in Fura-2-loaded HEK293 expressing TRPL-**F**467**Y** in response to 40 μM ETYA (**c**) or 40 μM ETI (**d**); TRPL-**I**465**V** in response to 40 μM ETYA (**e**) or 40 μM ETI (**f**) compared to TRPL-WT. Mean traces ± SEM are shown. Statistical analyses of data obtained during at least three independent experiments are presented as bar graphs (**g**). The data are the difference of normalized ETI-ETYA signal amplitudes. Two-dimensional schematic representation of the TRPL channel in the membrane with the amino acids **I**465**I** and **S**471**M** in red that allow a distinction between ETI and ETYA response (**h**). Number of cells analyzed: **V**438**I**, *n* = 303; **G**440**A**, *n* = 248; **V**446 **L**, *n* = 243; **L**458**I**, *n* = 175; **M**461 **L**, *n* = 393; **I**465**V**, *n* = 398; **F**467**Y**, *n* = 236; **S**471 **M**, *n* = 274; **L**472**F**, *n* = 289; **L**479**C**, *n* = 271; **A**521**G**, *n* = 369; **N**522 **M**, *n* = 252; **I**745**V**, *n* = 354. Substituted amino acid residues are marked in bold

The mutation TRPL-F467Y exhibited a reduced response to both ETYA (Fig. 6a) and another PUFA, i.e., ETI (Fig. 6b), by approximately 70% compared to the TRPL-WT (Fig. 6c, d). However, whereas the TRPL-I465V mutation was blunted by about 70% following stimulation by ETYA (Fig. 6e), it fully remained responsive to ETI (Fig. 6f). This observation may be taken as a first indication that I465 allow discrimination between different PUFAs. To identify additional aa residues differentiating between ETYA vs. ETI stimulation, we examined the effects of both PUFAs on 13 mutants (Fig. 6g). In addition to mutation I465V, we found S471M displaying a reduced response to ETYA in comparison to ETI (Fig. 6g). Thus, these two mutations (I465V, S471M), which are located within transmembrane segment S3, are predicted to allow discrimination of the channel sensitivity towards activation by the two PUFAs: ETYA and ETI (Fig. 6h).

### Characterization of the modified TRP channel

**Fig. 7:**
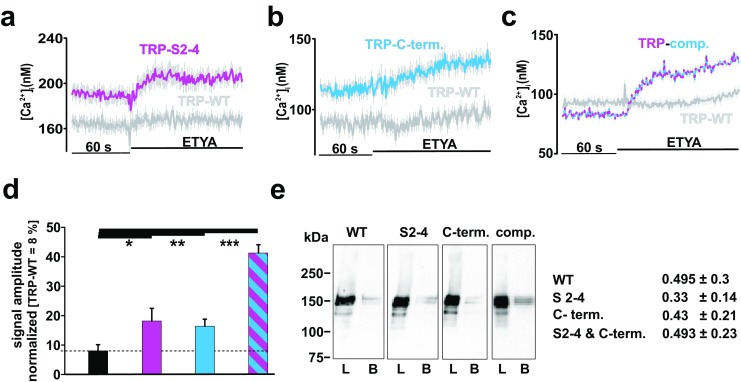
Functional characterization of the generated TRP variants. Changes in intracellular calcium concentration over time in Fura-2-loaded HEK293 in response to application of 40 μM ETYA in generated TRP variants (**a**–**c**). Mean traces ± SEM are shown. Statistical analysis of the normalized (TRPL-WT = 100%) signal amplitudes ± SEM (**d**) representing, in total, TRP-WT, *n* = 108, black; TRP-S2–4, *n* = 113 yellow; TRP-C terminal, *n* = 112, blue; and TRP-complete, *n* = 223, yellow-blue) (*p ≤ 0.05; **p ≤ 0.01; *p* ≤ 0.001). Western blot analysis (*n* = 4) of cell surface biotinylation, including the quantification of the band intensities ± SEM (**e**)

ETYA application did not increase the free intracellular Ca^2+^ levels in PUFA-insensitive TRP-WT channels (Fig. 7a–c, gray traces). In contrast, ETYA raised the cytosolic Ca^2+^ concentrations in a TRP mutant, where the identified residues in the transmembrane S2–S4 domains were replaced by TRPL counterparts required for PUFA sensitivity (see Fig. 7a, yellow trace). Similar results were obtained with a TRP variant where correspondingly cytosolic aa have been exchanged (see Fig. 7b). Cells expressing a TRP variant containing putative PUFA-sensitive residues in both the transmembrane and the proximal C-terminal stretches also increased intracellular Ca^2+^ concentration upon ETYA stimulation (see Fig. 7c). The statistical analysis of the normalized signal amplitudes showed that the ETYA effects on the TRP channel mutant containing PUFA-sensitive residues in the transmembrane and in the cytosolic C-terminal regions were more prominent as compared to the mutants carrying substitutions in the transmembrane or cytosolic regions only (Fig. 7d). For the latter the differences in response to ETYA correspond to 20% of the response of TRPL-WT (see Fig. 7d) whereas the fully modified TRP variant displayed 40% of the response of TRPL-WT, indicating that both regions are important for ETYA sensitivity.

In order to exclude differences in the membrane insertion of TRP mutants, we used a comparative cell surface biotinylation assay (Fig. 7e). Quantification of target proteins showed no differences in the channel insertion of the respective channels into the cell membrane (see Fig. 7e).

### Location of aa residues responsible for PUFA sensitivity in TRPL

**Fig. 8:**
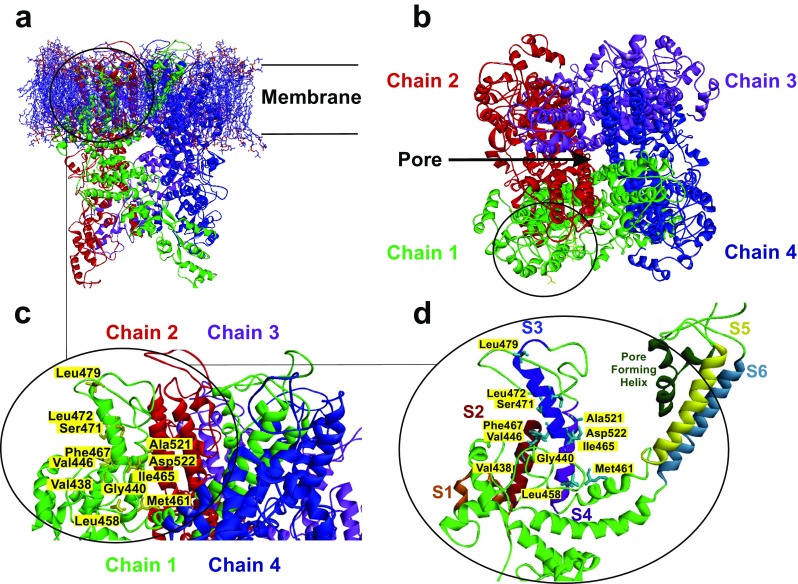
Tridimensional model of tetrameric TRPL channel. Full homology model viewed parallel to the membrane is shown **a**. View from the extracellular side (**b**), the four monomeric TRPL proteins forming the ion channel complex are colored: green (chain 1), red (chain 2), purple (chain 3), and blue (chain 4). The membrane is displayed with blue and red sticks, which illustrate the properties of the atom. The region of interest is zoomed in showing amino acid residues facing to membrane that are involved into the PUFA-induced activation of TRPL channel (**c**). Further magnified chain 1 shows location of these amino acids in the S2, S2–S3 linker, S3, and S4 segments (**d**). S1 segment is colored in brown, S2 dark red, S3 dark blue, S4 purple, S5 yellow, and S6 marine blue

By using a previously published approach, we constructed a 3D model of a *Drosophila* TRPL channel (Riehle et al. 2016). According to our model, each subunit consists of six transmembrane alpha-helices (S1–S6) that span the lipid bilayer, whereas the identified aa positions essential for the PUFA-sensitivity are within the transmembrane region (Fig. 8a). Furthermore, the TRPL channel has a tetrameric architecture in which subunits are arranged in fourfold symmetry around a central ion permeation path (Fig. 8b). aa residues essential for the PUFA-sensitivity are located on the lipid-facing periphery of the channel facing the membrane (Fig. 8c). These residues are mainly situated in the S2, the S2–S3 linker, S3, and S4 segments (Fig. 8d).

## Discussion

In this study, by exchanging aa between the PUFA-insensitive TRP and the PUFA-sensitive TRPL, we identified aa positions required for the activation of *Drosophila* TRP channels by PUFAs. These positions are located in transmembrane segments S2–S4 and in the proximal cytosolic C-terminus. We also identified two aa positions (465, 471) that allow discriminating the channel activating effects between two different PUFAs, i.e., the ETYA and ETI.

### aa positions localized in TRP transmembrane domains

We were able to identify 12 aa positions responsible for PUFA sensitivity in transmembrane segments S2–S4 (438, 440, 446, 458, 461, 465, 467, 471, 472, 479, 521, 522). The importance of the S2–4 domain was previously highlighted for the function of other subtypes of TRP channels (e.g., TRPC, TRPM, TRPV1) and voltage-dependent ion channels (e.g., K_V_7). According to widely accepted helix-packing models of voltage-sensitive ion channels, S1, S2, and S3 segments are located on the lipid-facing periphery of the tetrameric complex, whereas S5 and S6 consolidate the pore-forming channel core (Jordt and Julius 2002). In K_V_ channels, the positively charged aa in the S4 serve as voltage sensors and thereby play an important role in the channel’s function (Catterall 2011; Catterall and Swanson 2015). In line with these findings, we identified a crucial function of the S2–S4 region for the regulation of the *Drosophila* TRP channel superfamily. Our species-specific sequence comparison demonstrated a high conservation of all 18 identified aa positions within the PUFA-sensitive channels TRPL and TRPgamma. A large group of identified aa residues responsible for PUVA sensitivity in TRPL is located in the S2, the S2–S3 linker, S3, and S4 segments (Fig. 8d). Interestingly in TRPV1, capsaicin sensitivity is mediated by aa localized in the same S2–S4 area and largely dependent on R491, Y511, and S512 (Jordt and Julius 2002). While TRPV1 mutations in this region do not affect the temperature and pH sensitivity of the TRPV1 channel (Jordt and Julius 2002), they abolish activation of this channel by DAG (Woo et al. 2008). Of note, resiniferatoxin also activates TRPV1 through aa residuals M547 and T550 in S3 and S4 (Chou et al. 2004; Gavva et al. 2004). Cryo-EM micrographs of TRPV1 confirm these structure-activity relationships in the presence of vanilloids and revealed that the identified aa are located in a pocket formed by S3, S4, and the S4–S5 linker (Cao et al. 2013). S4–S5 linker is a critical constituent of TRPC4/C5 channel gating and that disturbance of its sequence allows channel opening (Beck et al. 2013). In addition, the S3–S4 region plays a central role in the activation of TRPV4 (Vriens et al. 2007). Thus, our and previous data indicate that the S2–S4 region represents a very ancient regulatory part of TRP channels that is responsible for channel activation by PUFA (Fig. 8).

### aa positions localized in the C-terminus of TRP

We identified additional aa residues (six aa; 742, 745, 775, 792, 793, 794) determining PUFA sensitivity of *Drosophila* TRP channels. They are located in the cytosolic C-terminus of TRP channels near the “TRP-box,” which represents a unique aa sequence (IWRLQR) required for important features of TRP channel function. For example in the TRPV subfamily, the TRP-box participates in the multimerization of channel proteins (Garcia-Sanz et al. 2004; Valente et al. 2008). In TRPM6 and TRPM8, the TRP-box is essential for the pore opening and the activation of the channels (Xie et al. 2011; Taberner et al. 2015; Valente et al. 2008), e.g., via interaction with the S4–S5 linker (Taberner et al. 2014). Cryo-EM images of the TRPV2 channel suggest that TRP channel openings also involve interactions of S6 with the C-terminal TRP-box and the N-terminal ankyrine repeat (Zubcevic et al. 2016). Five aa residues in the TRP-box are responsible for sensitivity of TRPC3 channels to erythropoietin (Tong et al. 2008) leading to TRPC3 channel stimulation (Hirschler-Laszkiewicz et al. 2009; Hirschler-Laszkiewicz et al. 2011). Moreover, C-terminal regions of the TRPL contain phosphorylation sites that led to enhanced degradation of TRPL and partial mislocalization of the channels if mutated (Cerny et al. 2013). These findings suggest that aa residues of cytosolic C-terminal region are important structures for altering the transmembrane transport properties of TRP channels. Our data suggest that this region is also important for activation of *Drosophila* TRP channels by PUFA. In particular, our study revealed involvement of transmembrane and cytosolic C-terminal aa in PUFA-induced activation of TRPL.

### Pathophysiological consequences and clinical relevance

Our study may have important clinical implications. Mutations of residues important for PUFA-induced activation of TRP channels might lead to abnormal TRP channel function, excessive Ca^2+^ influx, and abnormal cell function or cell death. Until now, several GOF mutations within TRP subfamilies have been identified to cause human pathologies, which are related to abnormal PUFA signaling. A GOF point mutation in the transmembrane segment S4 of TRPA1 (N855S) causes the familial episodic pain syndrome (FEPS) (Kremeyer et al. 2010). The patients have an enhanced secondary hyperalgesia to punctate stimuli on treatment with mustard oil (Kremeyer et al. 2010). Of note, mustard oil contains different PUFAs (Velasco et al. 1997). Also, TRPV3 mutations in the linker region between S4 and S5 and in the TRP-box cause the Olmsted syndrome (congenital palmoplantar and periorificial keratoderma, alopecia, and itching), highlighting the importance of transmembrane and C-terminal domain for the function of TRP channels (Yoshioka et al. 2009; Lin et al. 2012). A GOF mutation in TRPV4 causes autosomal dominant brachyolmia with elevated channel activation by several agonists, such as PUFA, including arachidonic acid (Rock et al. 2008). Therefore, the abnormal PUFA sensitivity of TRP may represent a biochemical signaling pathway contributing to excess function of TRP channels contributing to human disease. Furthermore, GOF mutation in TRPC3 in the S4–S5 linker of the channel leads to ataxia (moonwalker mouse) (Becker 2014). The effect of GOF mutations of TRPC6 in the pathophysiology of human kidney diseases has been well documented by several independent groups (Winn et al. 2005; Reiser et al. 2005). The identification of missense mutations in TRPC6 (P112Q, R895C, and E897K) that increase TRPC6 activity in recombinant expression systems provided the molecular basis for linking TRPC6 mutations to familial FSGS (Winn et al. 2005; Reiser et al. 2005; Riehle et al. 2016). If PUFAs contribute to the enhanced activity of TRP channels, then PUFA restriction or the pharmacological block of PUFAs synthesis seems to be an attractive hypothesis to attenuate the progression of chronic kidney disease in patients with GOF mutations of TPRC6 and familiar form of FSGS. On the other hand, PUFA supplementation should be performed in such patients with caution and undergo further clinical investigations. This treatment strategy is already under investigation in patients with chronic IgA nephropathy (Ferraro et al. 2009; Fassett et al. 2010).

### Mechanisms of PUFA actions

Arachidonic acid is one of the most frequently studied PUFA to exhibit various biological actions (for review, see Boland and Drzewiecki 2008; Elinder and Liin 2017). In those studies, ETYA has been often used as synthetic nonmetabolizable analogue of the arachidonic acid (Kehl 2001). The studies revealed that ETYA regulates many pharmacological targets in cells, including protein kinase C and several ion channels (Seifert et al. 1988; Meves 1994). ETYA is known to activate ether à go-go K^+^ channels (EAG) and to inhibit fast-inactivating (K_V_4) K^+^ channels (Gavrilova-Ruch et al. 2007; Ramakers and Storm 2002; Villarroel and Schwarz 1996). Moreover, ETYA can inhibit TRPM8 channels and activates TRPgamma (Andersson et al. 2007; Jors et al. 2006). This may indicate that the type of ETYA action is different depending to which (sub)family the respective channel belongs possibly involving differences in the molecular structure of the channels. Although putative binding site(s) within these channels for PUFAs are still a matter of intensive debate, there is data indicating that human ClC-2 channels express specific binding sites for ETYA in the channel protein (Cuppoletti et al. 2017). This makes it feasible that there are PUFA-binding sites also in other channels, i.e., TRP channels, as indicated by our study.

Our study identified various aa residues necessary for PUFA-induced channel stimulation by combining site-directed mutagenesis with a functional read-out. Based on these findings, future studies should dissect residues important for PUFA-binding from those mediating the PUFA activation within the channels. Although we used supramaximal PUFA concentrations, dose-response studies of ETYA and ETI on mutated TRP channels should confirm our findings since we have not checked the PUFAs for biphasic dose-response curves in the mutants. In addition, TRP channel activity was measured rather indirectly using fluorescence-based calcium imaging. Voltage dependency and the time course of TRP channel activation by PUFAs have not been studied. However, our previous work using electrophysiological techniques clearly indicates that TRP channels can be activated by ETYA over a broad range of membrane voltage (Jors et al. 2006).

## Conclusions

By characterizing TRPL single mutants, we identified 18 aa positions that are essential for the activation of TRPL by two PUFAs, namely ETYA and ETI. In contrast to other subtypes of TRP channels (e.g., TRPC, TRPM, TRPV1) and voltage-dependent ion channels (e.g., K_V_7), these positions are located not only in transmembrane segments S2–S4 but also in the cytosolic C-terminus. The additive involvement of transmembrane and cytosolic C-terminal aa in the recognition and signaling of PUFAs is a unique regulatory property of *Drosophila* TRP channels and has important pathophysiological implications for TRP channels exhibiting PUFA-regulatory sites or acquire them during mutational processes in human pathologies.

## Electronic supplementary material

ESM 1 (PDF 245 kb)
