# Supplementary material for: Molecular basis for the sensitivity of TRP channels to polyunsaturated fatty acids
Source: Naunyn Schmiedebergs Arch Pharmacol. 2018 May 8;391(8):833–46. doi: 10.1007/s00210-018-1507-3 (PMC6061713; doi:10.1007/s00210-018-1507-3)
Supplement: Supplementary file 1 — (PDF 245 kb) [file 210_2018_1507_MOESM1_ESM.pdf]

Supplementary Material

Molecular basis for the sensitivity of TRP channels to polyunsaturated fatty acids

Marc Riehle[1#], Dmitry Tsvetkov[1,2,3#], Björn-Oliver Gohlke[4], Robert Preissner[4], Christian Harteneck[1*§], Maik Gollasch[2,3§], Bernd Nürnberg[1§]

[1]Department of Pharmacology and Experimental Therapy, Institute of Experimental and Clinical Pharmacology and Toxicology, Eberhard Karls University Hospitals and Clinics, and Interfaculty Center of Pharmacogenomics and Drug Research, Wilhelmstrasse 56, 72074 Tübingen, Germany

[2]Experimental and Clinical Research Center (ECRC), a joint cooperation of the Charité University Medicine and Max Delbruck Center for Molecular Medicine in the Helmholtz Association, Lindenberger Weg 80, 13125 Berlin, Germany

[3]Medical Clinic for Nephrology and Internal Intensive Care, Charité Campus Virchow Klinikum

[4]Structural Bioinformatics Group, Institute for Physiology, Charité - University Medicine Berlin, Berlin, Germany.

# shared first authors, [§]shared senior authors, *deceased

Correspondence to:
Bernd Nürnberg, MD, PhD, bernd.nuernberg@uni-tuebingen.de or Maik Gollasch, MD, PhD, maik.gollasch@charite.de

Supplementary Table 1 Oligonucleotides used for targeting mutagenesis of TRPL (multiple mutants)

Mutation	Primer	Sequence
TRPL-GQ367-386AK	Sense	atgtgcgccccgaattgccggacggCcAagttgatgcgcaagccctttatgaa
TRPL-GQ367-386AK	Antisense	ttcataaagggcttgcgcatcaactTgGccgtccggcaattcggggcgcacat
TRPL-VIG438IIA	Sense	ctcatcgtggtgatgtacAtCattgCgttcgtgtgggaggaggt
TRPL-VIG438IIA	Antisense	acctcctcccacacgaacGcaatGaTgtacatcaccacgatgag
TRPL-WEEV443FEEL	Sense	acgtgattgggttcgtgtTCgaggagCtCcaggagatatttgccgtg
TRPL-WEEV443FEEL	Antisense	cacggcaaatatctcctgGaGctcctcGAacacgaacccaatcacgt
TRPL-LRNM458IRNL	Sense	atttgccgtgggcatgaagagttatAtCcgGaacCtgtggaacttcatcgactttctgc
TRPL-LRNM458IRNL	Antisense	gcagaaagtcgatgaagttccacaGgttCcgGaTataactcttcatgcccacggcaaat
TRPL-IDFLRNSL465VDYLRNMF	Sense	tatttgcgcaacatgtggaacttcGtcgactAtctgcgcaacaTGTtctacgtgagtgtgatgtgtttaa
TRPL-IDFLRNSL465VDYLRNMF	Antisense	ttaaacacatcacactcacgtagaACAtgttgcgcagaTagtcgaCgaagttccacatgttgcgcaaata
TRPL-SL471MF	Sense	gaacttcatcgactttctCcgGaacaTGTtctacgtgagtgtgatgtgt
TRPL-SL471MF	Antisense	acacatcacactcacgtagaACAtgttCcgGagaaagtcgatgaagttc
TRPL-QQATEE486IQATD	Sense	tgtttaagagcctttgcctacatcATCcaggccacagaCatCgcGagggatccgcagatggcttacata
TRPL-QQATEE486IQATD	Antisense	tatgtaagccatctgcggatccctCgcGatGtctgtggcctgGATgatgtaggcaaaggctcttaaaca
TRPL-AN521GM	Sense	cgaaggtctctttgcggcCgGcaTGgtcttctcggccttgaag
TRPL-AN521GM	Antisense	cttcaaggccgagaagacCAtgCcGgccgcaaagagaccttcg
TRPL-IAEGL513LAEGA	Sense	gattttgacccgcaactaCtagccgaaggCGCctttgcggcggccaatgt
TRPL- IAEGL513LAEGA	Antisense	acattggccgccgcaaagGCGccttcggctaGtagttgcgggtcaaaatc
TRPL-RSK729SSM	Sense	gtctagcaagaccatcgatcgtcaaTCctccaTgaaacgaaaggagcaggagcagttta
TRPL-RSK729SSM	Antisense	aactgctcctgctcctttcgtttcAtggagGAttgacgatcgatggtcttgctagactt
TRPL-YDNI742HDNV	Sense	caggagcagtttagcgagCatgataaCGtTatgcgctccctggtttgg
TRPL- YDNI742HDNV	Antisense	ccaaaccagggagcgcatAaCGttatcatGctcgctaaactgctcctg
TRPL-KE760RD	Sense	tggccgccatgcatcgcaGgttcgaCaataatcccgtatcggag
TRPL-KE760RD	Antisense	ctccgatacgggattattGtcgaacCtgcgatgcatggcggcca
TRPL-SGM792NSW	Sense	atgctcgagatattcgagaacaAcAgCTGggatgtttcctcggccaacaaaaagg
TRPL-SGM792NSW	Antisense	cctttttgttggccgaggaaacatccCAGcTgTtgttctcgaatatctcgagcat
TRPL-LM818-819IL	Sense	cggcgtatcaaagtgtgggaacggcgcAtTCtAaagggcttccaggtggcgccagtgcaaa
TRPL-LM818IL	Antisense	tttgcactggcgccacctggaagcccttTaGAaTgcgccgttcccacactttgatacgccg
TRPL-AP825GF	Sense	acggcgcttgatgaagggcttccaggtAgGATTCgtgcaaaatggctgtgagctcgatgcc
TRPL-AP825GF	Antisense	ggcatcgagctcacagccattttgcacGAATCcTacctggaagcccttcatcaagcgccgt

Supplementary Table 2 Oligonucleotides used for targeting mutagenesis of TRPL (single mutants)

Mutation	Primer	Sequence
TRPL-F389M	Sense	tgcctcctcctatttgttcttcctgAtGatcttgattctggtgtctcagcgag
TRPL-F389M	Antisense	ctcgctgagacaccagaatcaagatCaTcaggaagaacaaataggaggaggca
TRPL-L393A	Sense	tttgttcttcctgttcatcttgattGCggtgtctcagcgagcggacgatgact
TRPL-L393A	Antisense	agtcatcgtccgctcgctgagacaccGCaatcaagatgaacaggaagaacaaa
TRPL-F406L	Sense	cggacgatgactttgtacgcatcttGgggacgaccaggatgaagaaggagc
TRPL-F406L	Antisense	gctccttcttcatcctggtcgtcccCaagatgcgtacaaagtcatcgtccg
TRPL-L420K	Sense	aagaaggagctggcggagcaggagAAgcgtcagcgaggccagacacccagc
TRPL-L420K	Antisense	gctgggtgtctggcctcgctgacgcTTctcctgctccgccagctccttctt
TRPL-V438I	Sense	tggagctcatcgtggtgatgtacAtCattgggttcgtgtgggaggaggt
TRPL-V438I	Antisense	acctcctcccacacgaacccaatGaTgtacatcaccacgatgagctcca
TRPL-G440A	Sense	tcatcgtggtgatgtacgtgattgCgttcgtgtgggaggaggtgcagga
TRPL-G440A	Antisense	tcctgcacctcctcccacacgaacGcaatcacgtacatcaccacgatga
TRPL-W443F	Sense	acgtgattgggttcgtgtTCgaAgaggtgcaggagatattt
TRPL-W443F	Antisense	aaatatctcctgcacctcTtcGAacacgaacccaatcacgt
TRPL-V446L	Sense	tgggttcgtgtgggaggagCtCcaggagatatttgccgtg
TRPL-V446L	Antisense	cacggcaaatatctcctgGaGctcctcccacacgaaccca
TRPL-V452D	Sense	gcgaggtgaagcaactgtgggacgAtggCctgcaggagtatctcaacgacatgt
TRPL-V452D	Antisense	acatgtcgttgagatactcctgcagGccaTcgtcccacagttgcttcacctcgc
TRPL-L458I	Sense	tgccgtgggcatgaagagttatAtCcgcaacatgtggaacttcatcga
TRPL-L458I	Antisense	tcgatgaagttccacatgttgcgGaTataactcttcatgcccacggca
TRPL-M461L	Sense	gcatgaagagttatttgcgcaacTtgtggaacttcatcgactttctg
TRPL-M461L	Antisense	cagaaagtcgatgaagttccacaAgttgcgcaaataactcttcatgc
TRPL-I465V	Sense	cgcaacatgtggaacttcGtcgactttctgcgcaaca
TRPL-I465V	Antisense	tgttgcgcagaaagtcgaCgaagttccacatgttgcg
TRPL-F467Y	Sense	gcaacatgtggaacttcatcgactAtctgcgcaacagtctctacgtgagt
TRPL-F467Y	Antisense	actcacgtagagactgttgcgcagaTagtcgatgaagttccacatgttgc
TRPL-S471M	Sense	gaacttcatcgactttctCcgGaacaTGctctacgtgagtgtgatg
TRPL-S471M	Antisense	catcacactcacgtagagCAtgttCcgGagaaagtcgatgaagttc
TRPL-L472F	Sense	ttcatcgactttctgcgcaacagtTtctacgtgagtgtgatgtgtttaa
TRPL-L472F	Antisense	ttaaacacatcacactcacgtagaAactgttgcgcagaaagtcgatgaa
TRPL-L479C	Sense	acagtctctacgtgagtgtgatgtTAttaagagcctttgcctacatcca
TRPL-L479C	Antisense	tggatgtaggcaaaggctcttaaTAacatcacactcacgtagagactgt
TRPL-Q486I	Sense	gctttgcgggttgtatccttctttATTgtAcaaaaagagatgatatacaactcg
TRPL-Q486I	Antisense	cgagttgtatatcatctctttttgTacAATaaagaaggatacaacccgcaaagc
TRPL-E490D	Sense	atccttctttcaggtccaaaaagaTatgatatacaactcgcatgcgacgga
TRPL-E470D	Antisense	tccgtcgcatgcgagttgtatatcatAtctttttggacctgaaagaaggat
TRPL-A521G	Sense	tagccgaaggtctctttgcggcggGcaatgtcttctcggccttgaagct

TRPL-A521G	Antisense	agcttcaaggccgagaagacattgCccgccgcaaagagaccttcggcta
TRPL-N522M	Sense	ccgaaggtctctttgcggcggccaTGgtcttctcggccttgaagctggtgc
TRPL-N522M	Antisense	gcaccagcttcaaggccgagaagacCAtggccgccgcaaagagaccttcgg
TRPL-V550I	Sense	atctccctaggtcgcatgAtcatcgatatagtaaaat
TRPL-V550I	Antisense	attttactatatcgatgaTcatgcgacctagggagat
TRPL-K689Q	Sense	acggagtggaagtttgcccgaacccCagCtgtggatgagctatttcgaggaca
TRPL-K689Q	Antisense	tgtcctcgaaatagctcatccacaGctGggttcgggcaaacttccactccgt
TRPL-R729S	Sense	agtctagcaagaccatcgatcgtcaaTCctccaagaaacgaaaggagcaggag
TRPL-R729S	Antisense	ctcctgctcctttcgtttcttggagGAttgacgatcgatggtcttgctagact
TRPL-K731M	Sense	caagaccatcgatcgtcaacgctccaTgaaacgaaaggagcaggagcagttta
TRPL-K731M	Antisense	taaactgctcctgctcctttcgtttcAtggagcgttgacgatcgatggtcttg
TRPL-Y742H	Sense	aggagcaggagcagtttagcgagCaCgataatataatgcgctccctggt
TRPL-Y742H	Antisense	accagggagcgcattatattatcGtGctcgctaaactgctcctgctcct
TRPL-I745V	Sense	agcagtttagcgagtatgataatGtaatgcgctccctggtttggcga
TRPL-I745V	Antisense	tcgccaaaccagggagcgcattaCattatcatactcgctaaactgct
TRPL-V754I	Sense	ataatgcgctccctggtttggcgatatAtCgccgccatgcatcgcaagttcgagaat
TRPL-V754I	Antisense	attctcgaacttgcgatgcatggcggcGaTatatcgccaaaccagggagcgcattat
TRPL-V766I	Sense	caagttcgagaataatccGAtatcggaggacgatatca
TRPL-V766I	Antisense	tgatatcgtcctccgataTCggattattctcgaacttg
TRPL-N772I	Sense	atcccgtatcggaggacgatatcaTtgaggtcaagagtgagatcaatacga
TRPL-N772I	Antisense	cgtattgatctcactcttgacctcaAtgatatcgtcctccgatacgggatt
TRPL-K775R	Sense	cggaggacgatatcaatgaggtcaGgagtgagatcaatacgatgcgtta
TRPL-K775R	Antisense	taacgcatcgtattgatctcactcCtgacctcattgatatcgtcctccg
TRPL-S792N	Sense	agatgctcgagatattcgagaacaAcggaatggatgtttcctcggccaa
TRPL-S792N	Antisense	ttggccgaggaaacatccattccgTtgttctcgaatatctcgagcatct
TRPL-G793S	Sense	atgctcgagatattcgagaacagcTCaatggatgtttcctcggccaaca
TRPL-G793S	Antisense	tgttggccgaggaaacatccattGAgctgttctcgaatatctcgagcat
TRPL-M794W	Sense	tcgagatattcgagaacagcggaTGggatgtttcctcggccaacaaa
TRPL-M794W	Antisense	tttgttggccgaggaaacatccCAtccgctgttctcgaatatctcga
TRPL-G821D	Sense	agtgtgggaacggcgcttgatgaaggActtccaggtggcgccagtgcaaaatg
TRPL-G821D	Antisense	cattttgcactggcgccacctggaagTccttcatcaagcgccgttcccacact

Supplementary Table 3 Oligonucleotides used for targeting mutagenesis of TRP

Mutation	Primer	Sequence
TRP-IMA431VMG	Sense	ATTGAACTGGCAATCATTACCTATgTAATGGgTCTAATATTTGAGGAACTGAAATCT
TRP-IMA431VMG	Antisense	AGATTTCAGTTCCTCAAATATTAGAcCCATTAcATAGGTAATGATTGCCAGTTCAAT
TRP-L439V	Sense	ATAATGGCTCTAATATTTGAGGAAgTGAAATCTTTATATTCGGACGGCT
TRP-L439V	Antisense	AGCCGTCCGAATATAAAGATTTCAcTTCCTCAAATATTAGAGCCATTAT
TRP-L454M	Sense	ACGGCTTGTTTGAGTACATCATGGATaTgTGGAACATAGTGGACTACATATCGA
TRP-L454M	Antisense	TCGATATGTAGTCCACTATGTTCCAcAtATCCATGATGTACTCAAACAAGCCGT
TRP-V458I	Sense	AGTACATCATGGATCTTTGGAACATAaTtGACTACATATCGAACATGTTCTAT
TRP-V458I	Antisense	ATAGAACATGTTCGATATGTAGTCaAtTATGTTCCAAAGATCCATGATGTACT
TRP-MF464SL	Sense	ACATAGTGGACTACATATCGAACAgccTCTATGTGACGTGGATTCTTTGTAGG
TRP-MF464SL	Antisense	CCTACAAAGAATCCACGTCACATAGAggcTGTTCGATATGTAGTCCACTATGT
TRP-C472L	Sense	ACAgtcTCTATGTGACGTGGATTCTTTtaAGGGCCACCGCTTGGGTAATCGTCCATC
TRP-C472L	Antisense	GATGGACGATTACCCAAGCGGTGGCCCTtaAAAGAATCCACGTCACATAGAgacTGT
TRP-GM514AN	Sense	TATCAGAGGGCGCCTTTGCTGCCGcAAacGTCTTCTCCTATCTAAAGCTCGTC
TRP-GM514AN	Antisense	GACGAGCTTTAGATAGGAGAAGACgtTTgCGGCAGCAAAGGCGCCCTCTGATA
TRP-H734Y	Sense	AGTCCATGGAACGGGCACAGACGCTGtATGACAAAaTcATGAAGCTGCTGGTC
TRP-H734Y	Antisense	GACCAGCAGCTTCATgAtTTTGTCATaCAGCGTCTGTGCCCGTTCCATGGACT
TRP-V737I	Sense	ACGGGCACAGACGCTGCATGACAAAaTcATGAAGCTGCTGGTCAGGAGGTACA
TRP-V737I	Antisense	TGTACCTCCTGACCAGCAGCTTCATgAtTTTGTCATGCAGCGTCTGTGCCCGT
TRP-R767K	Sense	ATTACCGAGGATGATATCATTGAGGTGaagCAGGACATCAGCTCCTTGCGGTTCGAG

TRP-R767K	Antisense	CTCGAACCGCAAGGAGCTGATGTCCTGcttCACCTCAATGATAT CATCCTCGGTAAT
TRP-NSW784SGM	Sense	GAGTTGCTGGAGATTTTCACCAACAgTgGCatGGATGTACCCGA CATTGAGAAGAAG
TRP-NSW784SGM	Antisense	CTTCTTCTCAATGTCGGGTACATCCatGCcAcTGTTGGTGAAAAT CTCCAGCAACTC

Supplementary Table 4 Gene Bank numbers for TRP channels from different species

**Channel**	**Specie**	**Trivial name**	**Gen Bank Number**
TRP	*Aedes aegypti*	yellow fever mosquito	XP_001650890
TRP	*Bombus impatiens*	common eastern bumble bee	XP_003489572
TRP	*Culex quinquefasciatus*	southern house mosquito	XP_001869048
TRP	*Danaus plexippus*	monarch butterfly	EHJ65374
TRP	*Harpegnathos saltator*	Jerdon's jumping ant	EFN82264
TRP	*Musca domestica*	house fly	XP_005183118
TRP	*Nasonia vitripennis*	jewel wasp	XP_001605329
TRP	*Periplaneta americana*	American cockroach	AGG86916
TRP	*Tribolium castaneum*	red flour beetle	XP_968670
TRPL	*Bombus impatiens*	common eastern bumble bee	XP_003490000
TRPL	*Bombyx mori*	domestic silkworm	XP_004922702
TRPL	*Ceratitis capitata*	mediterranean fruit fly	XP_004517522
TRPL	*Culex quinquefasciatus*	southern house mosquito	XP_001847164
TRPL	*Harpegnathos saltator*	Jerdon's jumping ant	EFN80329
TRPL	*Musca domestica*	house fly	XP_005190181
TRPL	*Nasonia vitripennis*	jewel was	XP_001604491
TRPL	*Periplaneta americana*	American cockroach	AGG86915
TRPL	*Tribolium castaneum*	red flour beetle	XP_968598
TRPgamma	*Aedes aegypti*	yellow fever mosquito	XP_001650221
TRPgamma	*Bombus impatiens*	common eastern bumble bee	XP_003490670
TRPgamma	*Bombyx mori*	domestic silkworm	XP_004927991
TRPgamma	*Ceratitis capitata*	mediterranean fruit fly	XP_004536237
TRPgamma	*Danaus plexippus*	monarch butterfly	EHJ68691
TRPgamma	*Harpegnathos saltator*	Jerdon's jumping ant	EFN85066
TRPgamma	*Musca domestica*	house fly	XP_005191407
TRPgamma	*Nasonia vitripennis*	jewel wasp	XP_001604587
TRPgamma	*Periplaneta americana*	American cockroach	AAR26327

Supplementary Table 5 Prediction characteristics and comparison with the prediction algorithm SNAP2 (https://rostlab.org/owiki/index.php/Snap2) depending on the conservative versus non-conservative classification and experimental results obtained in the study

**Position**	**Wildtype Amino Acid/ side chain class**	**Side chain polarity**	**Side chain charge (ph 7.4)**	**Variant Amino Acids/ side chain class**	**Side chain polarity**	**Side chain charge (ph 7.4)**	**Type of Substitution: Concervative/ Non-Conservative**	**Predicted Effect (snap2)**	**Score (snap2)**	**Expected Accuracy (snap2)**	**Observed exp. effect (our data)**
367	G/aliphatic	nonpolar	neutral	A/aliphatic	nonpolar	neutral	Conservative	effect	27	63%	no
368	Q/ amide	polar	neutral	K/basic	basic polar	positive	Non-conservative	neutral	-27	61%	no
389	F/aromatic	nonpolar	neutral	M/sulfur containing	nonpolar	neutral	Conservative	neutral	-52	78%	no
393	L/aliphatic	nonpolar	neutral	A/aliphatic	nonpolar	neutral	Conservative	neutral	-19	57%	no
406	F/aromatic	nonpolar	neutral	L/aliphatic	nonpolar	neutral	Conservative	neutral	-24	61%	no
420	L/aliphatic	nonpolar	neutral	K/basic	basic polar	positive	Non-conservative	neutral	-17	57%	no
438	V/aliphatic	nonpolar	neutral	I/aliphatic	nonpolar	neutral	Conservative	neutral	-6	53%	yes
440	G/aliphatic	nonpolar	neutral	A/aliphatic	nonpolar	neutral	Conservative	effect	21	63%	yes
443	W/aromatic	nonpolar	neutral	F/aromatic	nonpolar	neutral	Conservative	neutral	0	53%	no
446	V/aliphatic	nonpolar	neutral	L/aliphatic	nonpolar	neutral	Conservative	neutral	35	66%	yes
452	V/aliphatic	nonpolar	neutral	D/acid	acidic polar	negative	Non-conservative	neutral	-26	61%	no
458	L/aliphatic	nonpolar	neutral	I/aliphatic	nonpolar	neutral	Conservative	neutral	-82	93%	yes

461	M/sulfur-containing	nonpolar	neutral	L/aliphatic	nonpolar	neutral	Conservative	neutral	-54	78%	yes
465	I/aliphatic	nonpolar	neutral	V/aliphatic	nonpolar	neutral	Conservative	neutral	-78	87%	yes
467	F/aromatic	nonpolar	neutral	Y/aromatic	nonpolar	neutral	Conservative	effect	12	59%	yes
471	S/hydroxyl-containing	polar	neutral	M/sulfur-containing	nonpolar	neutral	Non-conservative	neutral	-23	61%	yes
472	L/aliphatic	nonpolar	neutral	F/aromatic	nonpolar	neutral	Conservative	neutral	-53	78%	yes
479	L/aliphatic	nonpolar	neutral	C/sulfur-containing	nonpolar	neutral	Conservative	neutral	-65	82%	yes
486	Q/amide	polar	neutral	I/aliphatic	nonpolar	neutral	Conservative	neutral	-57	78%	no
490	E/acid	acidic polar	negative	D/acid	acidic polar	negative	Conservative	effect	21	63%	no
513	I/aliphatic	nonpolar	neutral	L/aliphatic	nonpolar	neutral	Conservative	neutral	53	78%	no
517	L/aliphatic	nonpolar	neutral	A/aliphatic	nonpolar	neutral	Conservative	neutral	-8	53%	no
521	A/aliphatic	nonpolar	neutral	G/aliphatic	nonpolar	neutral	Conservative	neutral	-26	61%	yes
522	N/amide	polar	neutral	M/sulfur-containing	nonpolar	neutral	Non-conservative	effect	25	63%	yes
550	V/aliphatic	nonpolar	neutral	I/aliphatic	nonpolar	neutral	Conserative	neutral	-57	78%	no
689	K/ basic	basic polar	positive	Q/amide	polar	neutral	Non-conservative	neutral	-55	78%	no
729	R/basic	basic polar	positive	S/hydroxyl-containing	polar	neutral	Non-conservative	neutral	-7	53%	no
731	K/ basic	basic polar	positive	M/sulfur-containing	nonpolar	neutral	Non-conservative	neutral	-10	53%	no
742	Y/aromatic	polar	neutral	H/basic aromatic	basic polar	positive(10%)	Non-conservative	effect	38	66%	yes

						neutral(90%)					
745	I/aliphatic	nonpolar	neutral	V/aliphatic	nonpolar	neutral	Conservative	neutral	-89	93%	yes
754	V/aliphatic	nonpolar	neutral	I/aliphatic	nonpolar	neutral	Conservative	neutral	-48	72%	no
760	K/basic	basic polar	positive	R/basic	basic polar	positive	Conservative	neutral	-62	82%	no
762	E/acid	acidic polar	negative	D/acid	acidic polar	negative	Conservative	neutral	-32	66%	no
766	V/aliphatic	nonpolar	neutral	I/aliphatic	nonpolar	neutral	Conservative	neutral	-33	66%	no
772	N/amide	polar	neutral	I/aliphatic	nonpolar	neutral	Non-conservative	neutral	-70	82%	no
775	K/ basic	basic polar	positive	R/basic	basic polar	positive	Conservative	neutral	-48	72%	yes
792	S/hydroxyl-containing	polar	neutral	N/amide	polar	neutral	Conservative	neutral	-72	87%	yes
793	G/aliphatic	nonpolar	neutral	S/hydroxyl-containing	polar	neutral	Non-conservative	effect	39	66%	yes
794	M/sulfur-containing	nonpolar	neutral	W/aromatic	nonpolar	neutral	Non-conservative	effect	18	59%	yes
818	L/aliphatic	nonpolar	neutral	I/aliphatic	nonpolar	neutral	Conservative	neutral	-25	61%	no
819	M/sulfur-containing	nonpolar	neutral	L/aliphatic	nonpolar	neutral	Conservative	neutral	-41	72%	no
821	G/aliphatic	nonpolar	neutral	D/acid	acidic polar	negative	Non-conservative	neutral	-57	78%	no
825	A/aliphatic	nonpolar	neutral	G/aliphatic	nonpolar	neutral	Conserative	neutral	-71	87%	no
826	P/cyclic	nonpolar	neutral	F/aromatic	nonpolar	neutral	Non-conservative	neutral	-16	57%	no

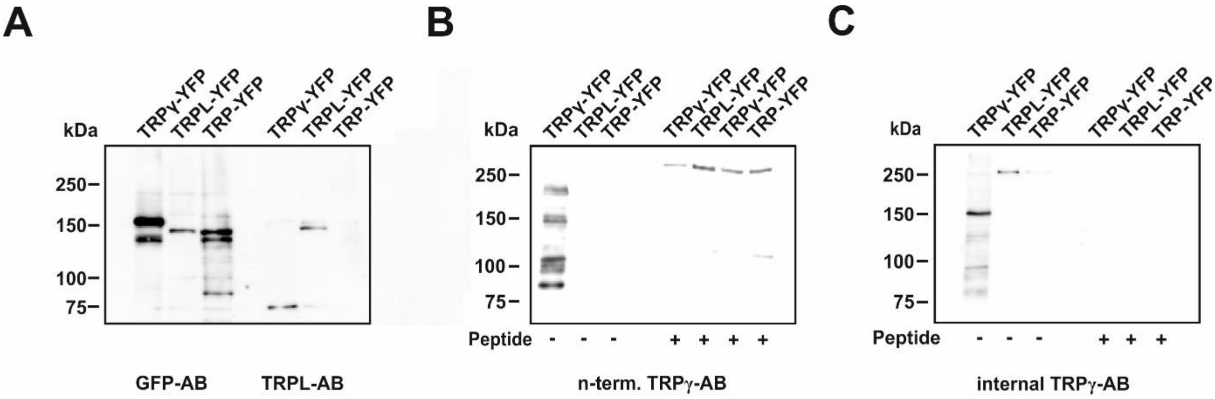

Supplementary Fig. 1

Detection of heterologously expressed *Drosophila* TRPC channels in HEK293 cells. Shown are exemplary Western blot analyzes of the protein extracts from transfected HEK293 cells. Demonstration of expression by means of a commercial GFP (A, left) and TRPL antibody (A, right). Detection of expression by means of a new n-terminal TRPgamma-AK (Pineda) (B, left) and by means of a new internal TRPgamma-AK (Pineda) (C, left), the specificity was confirmed by the immunogenic peptide (B and C, right).

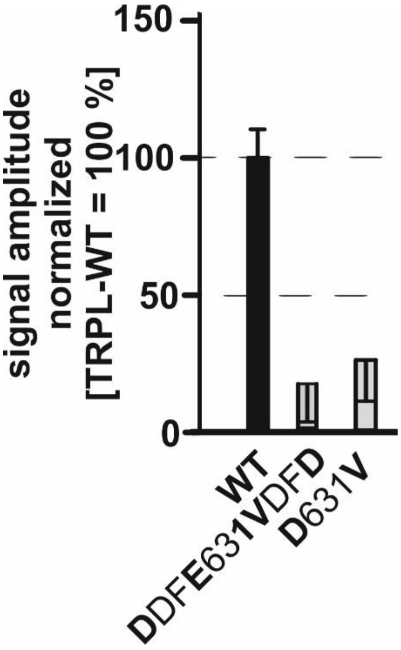

Supplementary Fig. 2

Validation of the approach for functional characterization of TRPL multiple and single mutants. Changes in intracellular calcium concentration over time in response to application of 40 µM ETYA in Fura-2–loaded HEK293 expressing TRPL-WT compared to TRPL mutants. Statistical analyses of data obtained during at least three independent experiments are presented as bar graphs. Data are means of the normalized (WT = 100%) signal amplitudes ± SEM representing the characterized mutants, in total, **D**DF**E**631**V**DF**D**, n = 46; **D**631**V**, n = 113. Substituted amino acid residues are marked in bold.
